# O Fascínio da Síndrome de Wolff-Parkinson-White

**DOI:** 10.36660/abc.20260471

**Published:** 2026-07-21

**Authors:** Pedro Brugada

**Affiliations:** 1 UZ Brussel Jette Bélgica UZ Brussel, Jette – Bélgica

**Keywords:** Síndrome de Wolff-Parkinson-White, Arritmias, Fibrilação Atrial

**Figura 1:**
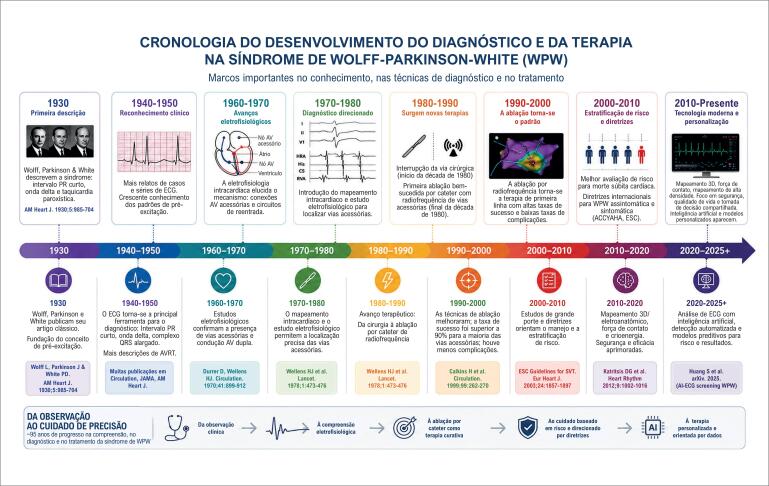
Cronologia do desenvolvimento do diagnóstico e da terapia na síndrome de Wolff-Parkinson-White.

Quase um século após sua descrição inicial,^1^ o número anual de publicações sobre a síndrome de Wolff-Parkinson-White (WPW) continua a aumentar (Tabela 1). Um fenômeno fascinante, considerando que já há alguns anos dispomos de técnicas para ablação e cura da doença. Poderia-se esperar uma atitude do tipo "problema resolvido" graças à ablação, e que o interesse pela doença diminuiria consideravelmente. A Figura 1 ilustra o progresso alcançado desde 1930. Cada nova publicação ainda traz informações importantes, como é o caso hoje do estudo de Mario et al.²

**Tabela 1:** Número estimado de publicações no PubMed

Período	Número estimado de publicações no PubMed
1950–1954	15
1955–1959	22
1960–1964	35
1965–1969	48
1970–1974	75
1975–1979	110
1980–1984	180
1985–1989	320
1990–1994	540
1995–1999	780
2000–2004	1050
2005–2009	1320
2010–2014	1640
2015–2019	1920
2020–2024	2350

Em uma grande coorte de pacientes com via acessória atrioventricular, os autores descobriram que idade avançada e localização lateral esquerda da via acessória estavam associadas a uma ocorrência significativa de fibrilação atrial espontânea, atingindo 18,6% no grupo etário acima de 60 anos, ou seja, quase um em cada cinco pacientes. Infelizmente, não havia dados disponíveis sobre a presença de vias acessórias manifestas versus ocultas.

Essas descobertas nos levam a uma questão muito importante em relação à prática, agora bastante difundida, de ablação da fibrilação atrial: os pacientes com fibrilação atrial são suficientemente estudados eletrofisiologicamente para excluir uma via acessória como causa da arritmia? Embora a ablação da via acessória tenha demonstrado reduzir a incidência de fibrilação atrial futura, a falha em identificar uma via acessória como causa da fibrilação atrial pode explicar o insucesso do isolamento das veias pulmonares na prevenção da fibrilação atrial em alguns pacientes. Uma via acessória oculta não será identificada sem um estudo eletrofisiológico completo. Acredito que esta seja a conclusão prática mais importante do excelente estudo de Mario et al.^2^
