# Supplementary material for: O Fascínio da Síndrome de Wolff-Parkinson-White
Source: Arq Bras Cardiol. 2026 Jul 21;123(5):e20260471. [Article in Portuguese] doi: 10.36660/abc.20260471 (PMC13399058; doi:10.36660/abc.20260471)
Supplement: Translation

# Translation: The Fascination of the Wolff-Parkinson-White Syndrome

**DOI:** 10.36660/abc.20260471i

**Authors:** Pedro Brugada

**Affiliations:** 1 Jette Belgium UZ Brussel, Jette – Belgium

**Keywords:** Wolff-Parkinson-White Syndrome, Arrhythmias, Atrial Fibrillation

Almost a century after its initial description,^1^ the yearly number of publications dealing with the Wolff-Parkinson-White syndrome (WPW) continues to increase (Table 1). A fascinating phenomenon if one considers that already for quite a few years, we have at our disposal techniques to ablate and cure the disease. One could expect an attitude like "problem solved" thanks to ablation, and that interest in the disease would markedly decrease. Figure 1 illustrates the progress made since 1930. Each new publication still brings important information, and so is today with the study by Souza Mario and coworkers.^2^

**Table 1:** Estimated number of PubMed publications

Period	Estimated number of PubMed publications
1950–1954	15
1955–1959	22
1960–1964	35
1965–1969	48
1970–1974	75
1975–1979	110
1980–1984	180
1985–1989	320
1990–1994	540
1995–1999	780
2000–2004	1050
2005–2009	1320
2010–2014	1640
2015–2019	1920
2020–2024	2350

**Figure 1:** A timeline of the development of diagnosis and therapy in Wolff-Parkinson-White syndrome.

In a large cohort of patients with an accessory atrio-ventricular pathway, the authors found that older age and a left lateral location of the accessory pathway were associated with a significant occurrence of spontaneous atrial fibrillation, reaching 18.6% in the age group older than 60 years, thus almost one in five patients. Regretfully, data on the presence of overt (manifest) versus concealed accessory pathways were not available.

These findings bring us to a very important question in relation to the now very extended practice of ablating atrial fibrillation: Are patients with atrial fibrillation sufficiently studied electrophysiologically to exclude an accessory pathway as the cause of arrhythmia? While ablation of the accessory pathway has been shown to reduce the incidence of future atrial fibrillation, failure to identify an accessory pathway as the cause of atrial fibrillation may explain the failure of pulmonary vein isolation to prevent atrial fibrillation in some patients. A concealed accessory pathway will not be identified without a complete electrophysiological study. I believe this is the most important practical conclusion from the excellent study by Souza Mario et al.^2^
